# High-frequency ultrasonography and contrast-enhanced ultrasound for the evaluation of testicular capillary hemangioma: A case report

**DOI:** 10.1097/MD.0000000000014779

**Published:** 2019-03-15

**Authors:** Ying He, Huimin Liao, Xi Xiang, Diming Cai, Li Qiu

**Affiliations:** Department of Ultrasound, West China Hospital, Sichuan University, Chengdu, China.

**Keywords:** contrast-enhanced ultrasound, high-frequency ultrasonography, testicular capillary hemangioma

## Abstract

**Rationale:**

Testicular capillary hemangioma (TCH) is a rare benign tumor of the testis. To the authors’ knowledge, there is currently only a few literatures describing the use of contrast-enhanced ultrasound (CEUS) to diagnose TCH. Accurate preoperative diagnosis of benign tumors can avoid orchiectomy. A case of TCH evaluated using high-frequency ultrasound and CEUS is presented.

**Patient concerns:**

A 21-year-old male presented with a right testicular mass during a routine physical examination, and was admitted to the authors’ hospital for definitive diagnosis and treatment.

**Diagnoses:**

Combined gray-scale, color Doppler ultrasonography, and CEUS did not exclude the possibility that the right testicular lesion may be a benign tumor. Combined with morphological and immunohistochemical staining results, a pathological diagnosis of TCH was considered.

**Interventions:**

The patient underwent right orchiectomy under general anesthesia, which proceeded smoothly.

**Outcomes:**

At the 12-month follow-up, the patient was completely asymptomatic and resumed all daily activities.

**Lessons:**

TCH is a rare benign tumor and lacks extensive previous data in imaging findings. If TCH can be diagnosed accurately before surgery, excessive or inappropriate treatment of benign lesions can be minimized, which will be beneficial to the physical and psychological health of patients.

## 1 Introduction

Testicular capillary hemangioma (TCH) is a rare benign tumor of the testis^[1]^; however, its etiology remains unclear. The incidence of TCH in children is higher than in adults.^[2]^ Although gray-scale and color Doppler ultrasound are widely used to characterize testicular lesions, they demonstrate low specificity in distinguishing benign and malignant intra-testicular solid lesions^[3]^ because the imaging manifestations of benign tumors are sometimes similar to those of malignant testicular lesions. Other advanced technologies, such as contrast-enhanced ultrasound (CEUS), have been suggested as adjunctive tools for testicular imaging.^[4]^ Some studies have reported that CEUS could improve the differential diagnosis of small testicular lesions.^[5]^ The use of CEUS provides more detail for the evaluation of testicular lesions. Presently, the literature pertaining to ultrasonography in the context of TCH is largely based on the description of gray-scale and color Doppler ultrasound.^[6–8]^ The literature on CEUS for TCH is scarce. This article describes a rare case of male TCH diagnosed using high-frequency ultrasonography and CEUS. The aim of the present report is to raise awareness of this disease.

## 2 Case report

Written informed consent for the publication of this case report and any accompanying images was obtained from the patient. Ethics approval for this study was waived by the Ethics Committee of West China Hospital, Sichuan University (Sichuan, China) because it involved fewer than 3 patients.

A lesion occupying the right testicle was discovered in a 21-year-old man during a routine medical examination. The patient presented with no abdominal pain, fever or gross hematuria. On physical examination, the patient's height and weight were normal. There was no breast development, and the size of the bilateral testicular morphology was normal. The patient had no history of malignant tumors, nor was there a family or genetic history. Laboratory tests revealed no abnormalities in alpha-fetoglobulin (AFP, 2.36 ng/mL [normal <8 ng/mL]), beta-human chorionic gonadotropin (β-HCG, 0.29 mIU/mL [normal <3.81 mIU/mL), carcinoembryonic antigen (0.93 ng/mL [normal <3.4 ng/mL]), free prostate-specific antigen (0.180 ng/mL [normal <0.75 ng/mL), and a normal level of the sex hormone testosterone (2.7 ng/mL [normal 2.49–8.36 ng/mL]). No abnormalities in routine blood, liver function, renal function, or electrolyte parameters were found.

**Figure 1:**
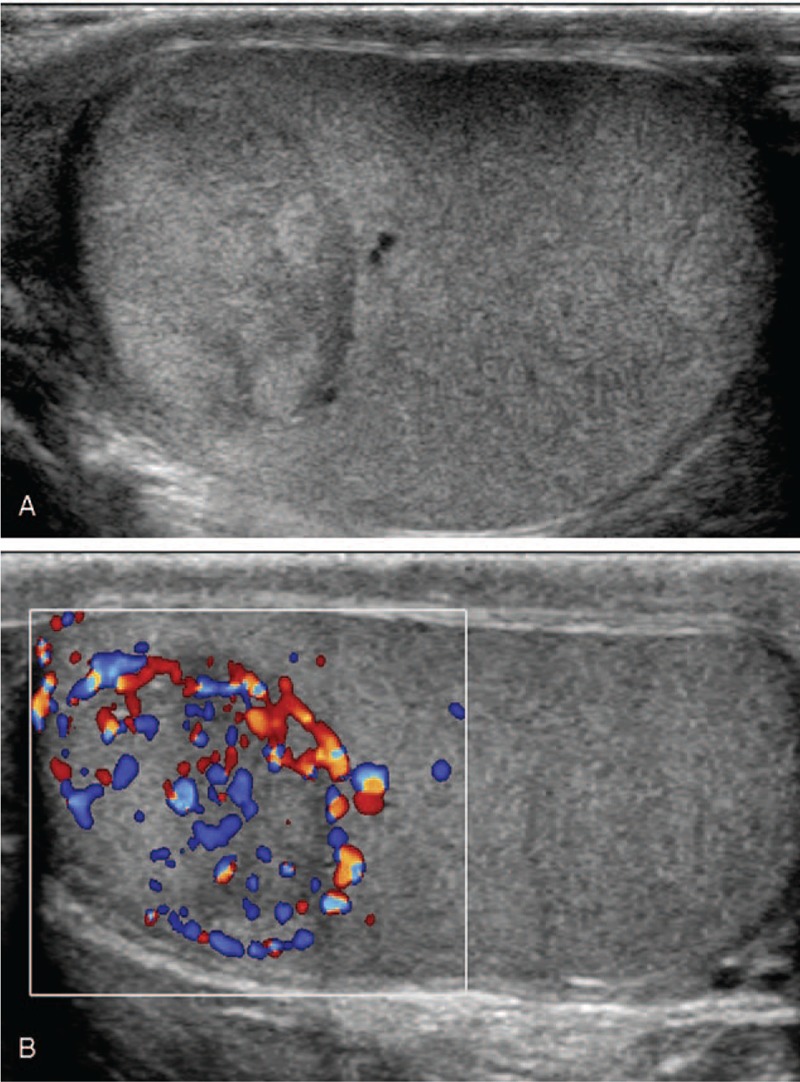
(A) Longitudinal image of the testis demonstrating a well-circumscribed, hypoechoic solid mass in the upper part of the testis. (B) Longitudinal color Doppler image of the testis demonstrating abundant blood flow signals in the mass.

The examinations were performed using an ultrasound device (Philips IU22, Philips Healthcare, Andover, MA) equipped with a 5 to 12 MHz linear-array transducer. High-frequency ultrasound detected a hypoechoic nodule, approximately 20 × 17 × 18 mm in size, in the upper portion of the right testis, with clear boundaries, regular shape, inhomogeneous internal echo, and abundant blood flow signals (Fig. 1A and 1B). There was no associated varicocele or hydrocele.

### 2.1 CEUS

**Figure 2:**
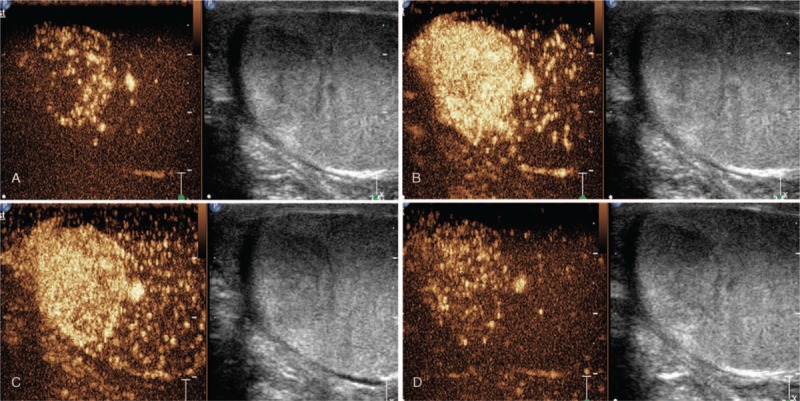
Longitudinal image of the testis. The left panel shows the CEUS mode, and the right shows the gray-scale mode. (A) CEUS image obtained 12 s after injection of microbubbles reveals a mass with peripheral enhancement. (B) The mass exhibits homogeneous enhancement at 22 s. (C) At 37 s, the contrast agent began to subside in a few areas. (D) The enhancement of microbubbles has diminished but is still more prominent than in the surrounding testicular area at 185 s. CEUS = contrast-enhanced ultrasonography.

Approximately 2.4 mL of acoustic contrast agent (SonoVue, Bracco, Milan, Italy) was injected through the median vein of the elbow, which was immediately followed by rapid injection of 10 mL of 0.9% saline. The distribution of contrast media and the change in echo intensity were observed continuously, and the enhanced image of the lesion was collected dynamically. Contrast agent began to appear in the periphery of the mass 12 s after injection, with obvious vascularization compared with the surrounding testicular tissue. The contrast agent exhibited clearly visible centripetal filling. The micro-bubbles filled rapidly and almost completely within 22 s. At 37 s, the contrast agent began to subside in a few areas. Although the enhancement of micro-bubbles diminished, it was still more prominent in the surrounding testicular tissue at 185 s (Fig. 2A–D). Combined gray-scale and color Doppler ultrasonography and CEUS findings strongly suggested that the possibility of a benign tumor could not excluded; however, the gold standard for definitive diagnosis was still dependent on pathology results.

**Figure 3:**
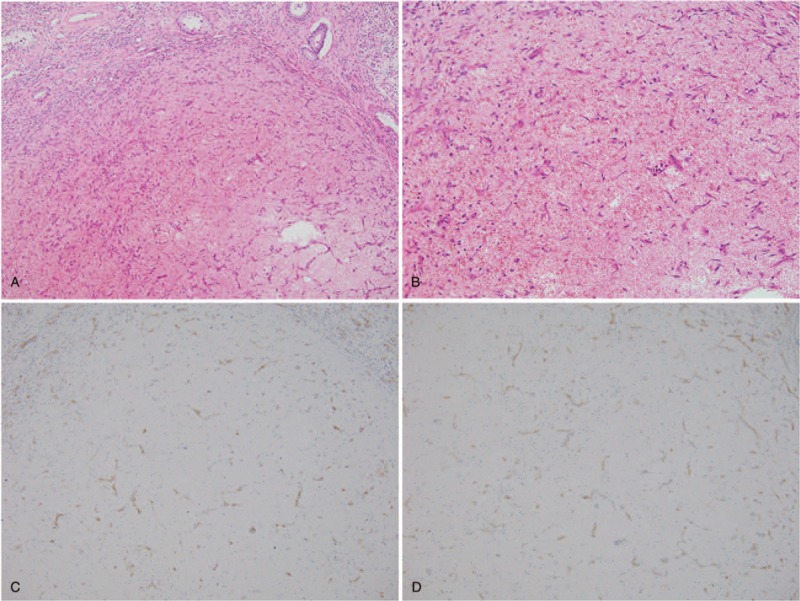
Hematoxylin-eosin staining revealing an abundant capillary network in the lesion. (A) Low-power view (original magnification × 100); (B): High-power view (original magnification × 200). Immunohistochemistry staining revealing a positive expression pattern: (C) CD31 (original magnification × 100), (D) CD34 (original magnification × 100).

The patient underwent right orchiectomy under general anesthesia. With the patient supine, an incision (approximately 5 cm in length) was made in the right groin, the outer ring mouth was separated layer by layer, the spermatic cord was completely ligated, and the affected testicle was removed. The operation proceeded smoothly. Pathological results revealed partial anastomosis of small vessel hyperplasia in the lesion, and no spermatogenesis cell components were found (Fig. 3A and B). Immunohistochemical staining demonstrated positive signals for CD31, CD 34 (Fig. 3C and D), erythroblast transformation-specific related gene and ki-67 (<1%), but negative signal for pan-cytokeratin and epithelial membrane antigen. Combined with morphological and immunohistochemical staining results, a pathological diagnosis of TCH was considered. At 12 months’ follow-up, the patient was completely asymptomatic and had resumed all daily activities.

## 3 Discussion

Most testicular tumors are malignant, and benign tumors are rare.^[9]^ Hemangioma is a benign tumor or vascular malformation resulting from the proliferation of vascular endothelial cells. The lesions are usually located in the head, face, trunk and limbs, and rarely located in the testis^[10]^; hence, TCH is very rare. Presently, only 26 articles have reported on TCH, with patient ages ranging from 10 months to 70 years. TCH occurs frequently in infants and children, but rarely in adults, with 63% of patients <18 years of age. Most of the patients were diagnosed by ultrasonography or physical examination. Seventy-three percent of patients exhibited no clinical symptoms, while only 27% experienced symptoms such as testicular pain. The lesion size ranges from 5 mm to 75 mm. Laboratory findings, including tumor markers and serum levels of AFP and β-HCG, were normal. Ultrasound was the first choice for imaging examination of TCH, and magnetic resonance imaging has been described in only 2 cases.^[11,12]^ On ultrasonography, 80% of patients exhibit a hypoechoic solid mass with abundant blood flow signals,^[2,6–8,13–16]^ with only 1 case exhibiting no significant increase in color Doppler flow signals.^[1]^ In addition to gray-scale and Doppler ultrasound, 1 patient underwent a 3-dimensional ultrasound scan to accurately visualize the vascular system of the lesion.^[17]^ CEUS and elastography were performed in another case, and the microvascular and tissue stiffness of the lesion were further characterized.^[18]^ In the literature, 63.6% of patients underwent total orchiectomy and 36.4% underwent partial orchiectomy. Postoperative pathology confirmed capillary hemangioma.

Ultrasound is the preferred imaging modality for testicular disease. Ultrasound imaging enables observation of the size, boundary, shape of the mass, and its relationship with surrounding tissues in real time and intuitively.^[19]^ The results of gray-scale and color Doppler ultrasonography in the present case revealed hypoechoic masses with abundant blood flow signals, consistent with that reported in the literature. As a new ultrasound technique, CEUS enables better and more detailed observation of the microcirculation of the lesion and can be performed as a supplementary modality to color Doppler ultrasound to further evaluate the lesion. To our knowledge, there is currently only 1 report in the literature describing the use of CEUS for testicular capillary tumors.^[18]^ The imaging modes described in the present case and the literature all demonstrate rapid enhancement in the arterial phase, slow clearance in the venous phase, and typical quick-in and slow-out. The enhancement pattern of contrast medium is consistent with that of CEUS of hemangiomas in other tissues and organs. CEUS of typical hemangiomas is characterized by nodular enhancement around the arterial phase, progressive centripetal filling, and hyperechoic/isoechoic changes in the portal and delayed phases.^[20–22]^

TCH is rare and has a low incidence. Given the scarcity of literature reports on the disease and lack of robust historical data in imaging diagnosis, TCH can be easily misdiagnosed as a malignant testicular tumor, such as seminoma, among others. On B-mode ultrasound, testicular seminoma typically appears as a homogeneous hypoechoic mass. On CEUS, the seminoma enhances faster than the surrounding normal testicular parenchyma, followed by rapid disappearance of the contrast agent in the lesion.^[23]^ According to Bhayana,^[24]^ differential diagnoses between benign lesions and metastases should be based on the time required for the elimination of microbubbles, which occurs significantly more rapidly in malignant lesions. Although the enhancement of micro-bubbles had diminished in our case, it was still more prominent than the surrounding testicular tissue at 185 s; therefore, it was concluded that the possibility of benign tumor could not excluded. As a supplementary examination method to gray-scale ultrasound, CEUS can augment the characteristics of the disease. CEUS is more sensitive and specific for the diagnosis of testicular non-neoplastic lesions and neoplastic lesions than gray-scale ultrasonography and elastography.^[25]^

Definitive diagnosis of TCH depends on histopathology. Histopathology reveals that the lesion is composed of abundant vascular lumen and small vessel hyperplasia. Immunohistochemically staining reveals positive expression of CD31, CD 34, and factor VIII, among others.^[26]^ Radical orchiectomy is still considered the standard treatment for testis masses of malignant or unknown origin.^[27]^ However, in recent years, testis-sparing surgery (TSS) has been increasingly used for the treatment of small testicular masses. Some authors have defined small testis tumors as <20 mm in diameter, ^[28]^ while others defined them as <15 mm.^[29]^ Tumor diameter appears to be one of the most important parameters for selected TSS indications. Some studies have demonstrated that TSS can be used to treat non-palpable asymptomatic testicular masses <20 mm in size^[30]^; the prevalence of benign histology is approximately 80%.^[31]^ Other authors have found that benign lesions are associated with a small diameter and suggested that <5 mm was the best threshold for predicting benign and malignant lesions.^[32]^ However, to date, there have been no randomized controlled trials comparing TSS with radical orchiectomy. Because the size of the lesion, in this case, was approximately 20 mm, we did not have sufficient evidence to confirm its benignity or malignancy before surgery; therefore, total orchiectomy was performed. Detailed preoperative physical examination, imaging findings, tumor markers and comprehensive evaluation of intraoperative frozen sections are helpful in the diagnosis of testicular masses, and decisions regarding treatment options and patient management. TCH is a benign neoplasm and, if the diagnosis can clearly be made before surgery, excessive/inappropriate treatment of benign lesions could be minimized, which in turn would be beneficial to the physical and psychological health of patients.^[33,34]^

## 4 Conclusion

TCH is very rare. Presently, it is difficult to diagnose before surgery due to the lack of robust data and experience in imaging findings. High-frequency ultrasound combined with CEUS can improve characterization and possibly explain imaging results for this disease.

## Author contributions

**Conceptualization:** Ying He, Huimin Liao, Xi Xiang, Diming Cai, Li Qiu.

**Investigation:** Huimin Liao.

**Project administration:** Xi Xiang.

**Resources:** Ying He, Diming Cai.

**Writing – original draft:** Ying He.

**Writing – review & editing:** Ying He, Diming Cai, Li Qiu.
